# Prevalence and risk factors associated with hypertension and prehypertension in a working population at high altitude in China: a cross-sectional study

**DOI:** 10.1186/s12199-017-0634-7

**Published:** 2017-04-04

**Authors:** Yang Shen, Chun Chang, Jingru Zhang, Ying Jiang, Bingying Ni, Yanling Wang

**Affiliations:** Department of Social Medicine and Health Education, School of Public Health, Peking University, Beijing, 100191 China

**Keywords:** Hypertension, Prehypertension, Risk factors, Workplaces, High altitude

## Abstract

**Background:**

Little information is available on the epidemiology of hypertension and prehypertension at high altitude in China, the aim of this study was to determine the prevalence of hypertension, prehypertension and their risk factors among Chinese working population at high altitude regions.

**Methods:**

A cross-sectional survey was performed in an occupational sample of 4198 employees aged 20–59 years on Qinghai-Tibet Plateau between May to July 2013. Information from a self-administered questionnaire, physical examinations and laboratory measurements were obtained from each participant. Multivariable analysis was performed to determine the association of various risk factors with hypertension and prehypertension.

**Results:**

The total crude prevalence of hypertension and prehypertension was 28.1 and 41.5%, respectively; the overall standardized prevalence of hypertension and prehypertension was 26.7 and 41.3%, respectively. Multivariate logistic regression showed that age, sex, ethnicity, job position, overweight or obesity, frequent drinking, family history of hypertension, diabetes and hyperuricemia were risk factors for hypertension, and age, sex, education, job position, overweight or obesity, current smoking and family history of hypertension were risk factors for prehypertension. Among the hypertensives, 36.5% were aware of their condition, 19.4% were being treated and 6.2% had their blood pressure (BP) controlled; among the treated hypertensives, 31.9% had their BP under control.

**Conclusions:**

There is a high prevalence of hypertension and prehypertension in the working population at high altitude in China, but with very low awareness, treatment and control rates. Workplace-based BP screening and intervention programs that aim to modify risk factors such as high BMI, tobacco use, alcohol consumption and inappropriate use of antihypertensive medicine are urgently needed.

**Electronic supplementary material:**

The online version of this article (doi:10.1186/s12199-017-0634-7) contains supplementary material, which is available to authorized users.

## Background

Cardiovascular disease (CVD) is the leading cause of death and disease burden globally, and hypertension (HTN), defined as systolic blood pressure (SBP) ≥140 mmHg and/or diastolic blood pressure (DBP) ≥90 mmHg, or taking antihypertensive medicine [1], is the most important preventable risk factor for CVD in China [2]. With the rapid economic development and urbanization of China, the prevalence of HTN has risen substantially. It is estimated that 325 million Chinese adults, 29.6% of the Chinese adult population, had HTN in 2010 [3]. However, this number does not include the prehypertension (PHT, representing a SBP of 120–139 mmHg and/or a DBP of 80–89 mmHg [1]), which is also identified as being independently associated with increased risk of HTN and CVD. Several national surveys reveal that PHT affects more than 20% of the general adult population, most commonly among middle-aged adults [4–6].

Most epidemiological studies have focused on the prevalence of HTN, PHT and their related risk factors in general Chinese population. However, little attention has been devoted to the occupational population, especially those working at high altitude areas. High altitude was considered as an altitude greater than 2400 m above the sea level [7], and exposure to hypoxia environment at high altitude is increasingly being identified as a risk factor for HTN [8]. For instance, a recent systematic review has indicated a 2% increase in the prevalence of HTN among the Tibetans with every 100 m increase in altitude in Tibet [9]. From an international perspective, the working population has been identified as the high-risk group for CVD due to the clustering of CVD risk factors such as raised blood pressure (BP), high BMI, and smoking [10–12]. There is a large number of workers on Qinghai-Tibet Plateau e.g., railroad construction, power maintenance, and their health status are closely related with the social stability and economic growth. Furthermore, the workplace is an ideal setting for health promotion as working population spend a considerable amount of their daily activity there [13]. Relevant information on the prevalence and determinants of HTN and PHT in the occupational samples at high altitude is crucial for establishing goals for effective workplace-based intervention programs.

The aim of the present study was to determine the prevalence of HTN and PHT and explore their potential risk factors in a convenience sample of young and middle-aged employees of 23 workplaces at China’s high altitude areas.

## Methods

### Study design and participants

The study was conducted among employees of a state-owned grid corporation in the Qinghai Province of China. This corporation was a special large-sized enterprise operating the business of construction, rehabilitation, operation and maintenance of power grid, and it was composed of 23 geographically separate subsidiaries located in Qinghai Province, which were the workplaces investigated in this study. Qinghai lies in western China - on the northeastern part of the Qinghai-Tibet Plateau with an average altitude above 3000 m, and the altitudes of the workplaces in our study follow a scale of 2600 to 3700 m. The corporation comprised approximately 6000 full-time employees at the time of survey, and the general manager of this corporation agreed to implement the investigation during the working hours and salary was not deducted for leaving work to participate in the survey. In total, 4975 employees who had been working at the corporation for at least 2 years were examined, and among them 777 employees were excluded from the study for failing to complete either a questionnaire survey, a mandatory physical examination, or a laboratory measurement. Therefore, 4198 employees aged 20-59 years were finally included, representing a convenience sample.

### Data collection

The data collection period was between May to July 2013. All subjects were asked to complete a self-administered, structured questionnaire in the meeting rooms of their workplaces. Clerical errors and blanks were checked by trained research staff and then corrected by subjects on-site. The questionnaire was divided into three main sections to obtain data on subjects’ demographic characteristics, health behaviors, and HTN-related information.

To be specific, in Section A (demographic characteristics), subjects’ age, sex, ethnicity (Han, Tibetan, or others), educational level (primary, middle, high school, college, or university and above) and job position (supervisor, or general staff) were asked. In Section B (health behaviors), subjects were asked “Do you currently smoke cigarettes?” (yes, one or more per day/yes, less than one cigarette per day/no, I quit smoking/I have never smoked) and “How often did you drink in the past month?” (daily/frequently (3 to 6 days a week)/occasionally (1 to 2 days a week)/did not drink any). In section C (HTN-related information) subjects were asked “Do any of your immediate family members (including parents, grandparents, and siblings) have HTN?” (yes/no), “Have you ever been told by a doctor or other healthcare professional that you had HTN?” (yes/no), and for those with HTN, they were also asked “Because of your HTN, are you now taking prescribed medicine?” (yes/no).

Measurements of BP, height and weight, and biochemical analysis of blood samples were all conducted in examination centers of designated hospitals. Subjects were suggested to avoid drinking, smoking, coffee, tea and exercise for at least 30 min prior to the measurement. BP measurements were taken after the subjects resting in a seated position quietly for at least 5 min. Throughout the survey, BP was measured three times at 5 min intervals in the morning. A previously validated electronic sphygmomanometer (Omron HEM-7201, Omron, Dalian, China) was used for the BP measurements in our study, this automated BP device has good accuracy and is recommended for use in adults at high altitudes [14]. The mean of the second and third readings defined a subject’s BP. Body height and weight were measured and body mass index (BMI) was then calculated as weight (kg) divided by height squared (m^2^). These indexes were all measured by trained physicians following standard protocols [15]. After an overnight fast of at least 8 h, the subjects’ venous blood samples were obtained by trained physicians to test the levels of total cholesterol (TC), triglycerides (TG), serum uric acid (SUA) and fasting blood glucose (FBG) in the laboratory.

### Definitions

Subjects were diagnosed with normotension (NTN), prehypertension (PHT) and hypertension (HTN) by trained physicians, according to the criteria from the Seventh Report of the Joint National Committee on Prevention, Detection, Evaluation, and Treatment of High Blood Pressure (JNC-7) [1]. HTN was defined as SBP ≥140 mm Hg and/or DBP ≥90 mm Hg, or current treatment with antihypertensive medications. PHT was defined as a SBP between 120 and 139 mm Hg and/or a DBP between 80 and 89 mm Hg. NTN was defined as SBP < 120 mm Hg and DBP < 80 mm Hg. BP Control was defined as an average SBP and DBP lower than 140/90mmHg while on treatment. Diabetes was defined as a FBG ≥7.0 mmol/L or a self-reported previous diagnosis of diabetes by the doctors. Based on the criteria of the 2007 Chinese Guidelines on Prevention and Treatment of Dyslipidemia in Adults [16], high TC was defined as total cholesterol ≥6.22 mmol/L and high TG as triglyceride ≥2.26 mmol/L. Subjects with BMI 24.0–27.9 kg/m^2^ was defined as overweight and BMI ≥28.0 kg/m^2^ as obesity, and overweight or obesity referred to a BMI ≥24.0 kg/m^2^ [17]. Criteria for hyperuricemia were SUA levels ≥357 μmol/L in women and 416 μmol/L in men, respectively [18]. Current smoking was defined as subjects who reported smoking either every day or some days at the time of survey. Frequent drinking was defined as subjects who reported drinking daily or frequently during the past 30 days preceding the survey.

### Statistical analysis

All continuous variables are presented as mean (SD), except for TC and TG, which are described by median (interquartile range) because of the skewed distribution. Categorical variables are presented as numbers (percentages). Prevalence rates in the overall population were calculated by the direct standardized method, according to the Sixth National Population Census of China (2010) [19]. One-way ANOVA was used to compare continuous variables with normal distribution among inter-group, and Student–Newman–Keuls (SNK) test was used for pairwise comparisons. Kruskal-Wallis test was used to compare continuous variables with non-normal distribution among inter-group and Wilcoxon rank-sum test was used for pairwise comparisons. Categorical variables were analyzed by Chi-squared test and Bonferroni adjustment was applied for pairwise comparisons in which a Bonferroni-adjusted *p-*value <0.05/3 or 0.017 was considered to be statistically significant. Multivariable logistic regression analyses were used to explore the risk factors of HTN (vs NTN) and PHT (vs NTN), with HTN and PHT serving as the dichotomous outcome variables, and sex, age, ethnicity, education, job position, overweight or obesity, current smoking, frequent drinking, family history of HTN, diabetes, hyperuricemia, high TC and high TG, as the candidates for inclusion in the analyses. All data analyses were conducted using SAS 9.4 (SAS institute, Cary, NC, USA) and a two-sided *p-*value <0.05 was considered statistically significant.

## Results

### Prevalence of HTN and PHT

Of the 4198 subjects, 3026 (72.1%) were men and 1172 (27.9%) were women, and the sex ratio (men: women) was 2.58:1. The mean (SD) age of the total was 39.77 (8.92) years, 39.75 (9.37) years for men and 39.82(7.62) years for women, respectively. Han Chinese were overrepresented and accounted for 90.4% of the total. Among all the subjects, 3545 (84.4%) were inhabitants and 653 (15.6%) were migrants from inland China, and the corresponding figures for Han Chinese were 3176 (83.7%) and 618 (16.3%), respectively. Overall, there were 1180 (28.1%) with HTN, 1744 (41.5%) with PHT, and 1274 (30.4%) with NTN. Age- and sex-standardized prevalence rates of HTN and PHT among subjects were 26.7% and 41.3%, respectively. The age-standardized prevalence of HTN and PHT was both higher in men than in women (30.8% vs 16.4%, 43.5% vs 36.1%, respectively).

**Fig. 1:**
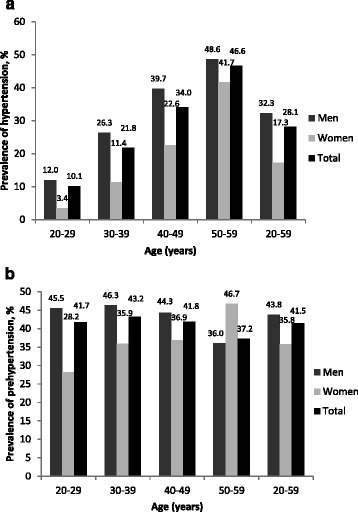
Age- and sex-specific prevalence of hypertension (**a**) and prehypertension (**b**) among subjects. The number of subjects according to the rising 10-year age group (20–59 years) in men were 615, 707, 1251, 453 and the corresponding figure in women were 174, 301, 637 and 60, respectively. The corresponding data for hypertension/prehypertension in men were 74/280, 186/327, 497/554 and 220/163, and those in women were 6/49, 34/108, 144/235 and 19/28

Figure 1 depicts the age-specific crude prevalence of HTN and PHT in men, women, and all subjects. Men had significantly higher prevalence of HTN and PHT compared with women (32.3% vs 17.3 and 43.8% vs 35.8%, respectively; *p* <0.001). The age-specific prevalence of HTN (Fig. 1a) increased distinctly with age in both men and women (*p* <0.001). In men, the prevalence age-specific of PHT (Fig. 1b) increased up to the age of 30 then decreased with age (*p* <0.001), whereas the opposite trend was observed in women participants (*p* <0.05). For each age group up to 50, men had a higher age-specific prevalence of PHT (Fig. 1b) than that of women, while the reverse pattern was observed in those older than 50 years, with women having greater age-specific prevalence of PHT.

### Characteristics of subjects according to BP status

Characteristics of subjects according to BP status is presented in Table 1. Men, Han ethnicity, those with high school education or less, supervisors, overweight or obese individuals, current smokers, frequent drinkers, diabetics and hyperuricemic increased significantly from NTN to PHT, and then eventually to HTN. There was an exception of this trend in the cases of individuals with high TG, higher degree, and those who had a family history of HTN. Moreover, there were significant positive relationships of age and anthropometric indices (BP, BMI, TC, TG, FBG, SUA), but inverse associations of medium education (those with diplomas or basic degrees) and general staffs with PHT and HTN.

**Table 1:** Characteristics of the subjects according to BP status

Characteristics	NTN (*n* = 1274)	PHT (*n* = 1744)	HTN (*n* = 1180)	*p*-value
Men (%)	725 (56.9)	1324 (75.9)^a^	977 (82.8)^a,b^	<0.001
Age (years)	37.42 (8.85)	40.20 (8.83)^a^	44.19 (7.84)^a,b^	< 0.001
Ethnicity (%)				
Han	1116 (87.6)	1579 (90.5)^a^	1099 (93.1)^a,b^	< 0.001
Other	158 (12.4)	165 (9.5)^a^	81 (6.9)^a,b^	< 0.001
Education (%)				
High school/less	218 (17.1)	394 (22.6)^a^	378 (32.0)^a,b^	< 0.001
Diploma/Basic degree	1015 (79.7)	1325 (76.0)^a^	786 (66.6)^a,b^	< 0.001
Higher degree	41 (3.2)	25 (1.4)^a^	16 (1.4)^a^	< 0.001
Job position (%)				
Supervisor	59 (4.6)	170 (9.8)^a^	223 (18.9)^a,b^	< 0.001
General staff	1215 (95.4)	1574 (90.2)^a^	957 (81.1)^a,b^	< 0.001
SBP (mmHg)	103.01 (7.01)	117.98 (8.28)^a^	131.82 (16.75)^a,b^	< 0.001
DBP (mmHg)	69.82 (4.79)	81.60 (3.81)^a^	94.25 (8.29)^a,b^	< 0.001
BMI (kg/m^2^)	21.94 (2.91)	23.54 (3.10)^a^	24.24 (3.26)^a,b^	< 0.001
TC (mmol/L)	4.19 (3.67, 4.81)	4.37 (3.79, 5.00)^a^	4.47 (3.90, 5.00)^a,b^	< 0.001
TG (mmol/L)	1.15 (0.83, 1.65)	1.37 (0.96, 2.09)^a^	1.60 (1.03, 2.23)^a,b^	< 0.001
FBG (mmol/L)	4.65 (0.80)	4.87 (1.17)^a^	5.00 (1.15)^a,b^	< 0.001
SUA (μmmol/L)	334.31 (86.23)	360.93 (84.57)^a^	378.76 (97.95)^a,b^	< 0.001
Overweight or Obesity (%)	283 (22.2)	748 (42.9)^a^	612 (51.9)^a,b^	< 0.001
High TC (%)	29 (2.3)	47 (2.7)	44 (3.7)	0.084
High TG (%)	153 (12.0)	352 (20.2)^a^	279 (23.6)^a^	< 0.001
Current smoking (%)	440 (34.5)	776 (44.5)^a^	612 (51.9)^a,b^	< 0.001
Frequent drinking (%)	70 (5.5)	170 (9.8)^a^	188 (15.9)^a,b^	< 0.001
Family history of HTN (%)	408 (32.0)	609 (34.9)	477 (40.4)^a,b^	< 0.001
Diabetes (%)	32 (2.5)	87 (5.0)^a^	109 (9.2)^a,b^	< 0.001
Hyperuricemia (%)	248 (19.5)	439 (25.2)^a^	407 (34.5)^a,b^	< 0.001

Data are presented as number (%), mean (SD) or median (range). For the multiple comparisons, Bonferroni adjustment, SNK test and Wilcoxon rank-sum test were used following the Chi-squared test, one-way ANOVA and Kruskal-Wallis test, respectively

*Abbreviations: NTN* normotension, *PHT* prehypertension, *HTN* hypertension, *BMI* body mass index, *SBP* systolic blood pressure, *DBP* diastolic blood pressure, *SUA* serum uric acid, *FBG* fasting blood glucose, *TC* total cholesterol, *TG* triglycerides

^a^An significant difference when compared to NTN

^b^An significant difference when compared to PHT

The prevalence rates of overweight or obesity, high TC, high TG, current smoking, frequent drinking, family history of HTN, diabetes and hyperuricemia among subjects were 39.1% (men: 45.7% vs women: 22.1%; *p* <0.001), 2.9% (men: 3.3% vs women: 1.7%; *p* <0.01), 18.7% (men: 22.5% vs women: 8.9%; *p* <0.001), 43.5% (men: 59.2% vs women: 3.2%; *p* <0.001), 10.2% (men: 13.8% vs women: 0.9%; *p* <0.001), 37.2% (men: 34.4% vs women: 44.5%; *p* <0.001), 5.4% (men: 6.7% vs women: 2.1%; *p* <0.001) and 26.1% (men: 29.6% vs women: 17.0%; *p* <0.001), respectively. [see Additional file 1].

### Risk factors associated with HTN and PHT

Table 2 shows the various risk factors associated with HTN and PHT by the multivariable logistic regression analysis. Men, higher age and job position, overweight or obesity, and family history of HTN were significantly associated with HTN and PHT. Han ethnicity, frequent drinking, diabetes and hyperuricemia were risk factors for HTN but were not for PHT. Compared to subjects with a high school or less level of education, those with a higher degree were less likely to have PHT. Current smoking was not significantly associated with HTN but was found to be negatively associated with PHT.

**Table 2:** Risk factors associated with HTN and PHT by multivariable logistic regression analysis

Variable	HTN		PHT	
	OR (95% CI)	*P*-value	OR (95% CI)	*p*-value
Sex (Men/Women)	1.779 (1.429–2.216)	< 0.001	2.505 (2.028–3.093)	< 0.001
Age group (years)				
20–29^a^	1		1	
30–39	2.272 (1.700–3.035)	< 0.001	1.429 (1.144–1.786)	0.002
40–49	3.976 (3.036–5.208)	< 0.001	2.016 (1.624–2.504)	< 0.001
50–59	5.395 (3.918–7.429)	< 0.001	2.057 (1.465–2.889)	< 0.001
Ethnicity (Han/Other)	1.393 (1.056–1.839)	0.023	1.245 (0.968–1.602)	0.088
Education				
High school/less^a^	1		1	
Diploma/Basic degree	0.837 (0.699–1.002)	0.060	0.891 (0.720–1.104)	0.291
Higher degree	0.610 (0.322–1.153)	0.135	0.436 (0.245–0.774)	0.005
Job position (Supervisor/General staff)	2.565 (2.051–3.208)	< 0.001	1.733 (1.247–2.408)	0.001
Overweight or obesity (Yes/No)	1.599 (1.368–1.868)	< 0.001	2.104 (1.762–2.513)	< 0.001
Current smoking (Yes/No)	0.977 (0.822–1.161)	0.800	0.773 (0.636–0.940)	0.010
Frequent drinking (Yes/No)	1.553(1.239–1.946)	< 0.001	1.248 (0.914–1.704)	0.163
Family history of HTN (Yes/No)	1.289 (1.106–1.503)	0.001	1.193 (1.009–1.410)	0.039
Diabetes (Yes/No)	1.474 (1.097–1.981)	0.010	1.214 (0.783–1.881)	0.386
Hyperuricemia (Yes/No)	1.635 (1.384–1.930)	< 0.001	1.097 (0.903–1.333)	0.353
High TC (Yes/No)	0.933 (0.612–1.421)	0.746	0.769 (0.454–1.300)	0.326
High TG (Yes/No)	1.094 (0.905–1.321)	0.352	1.198 (0.950–1.510)	0.127

*Abbreviations: OR* Odds ratio, *CI* Confidence interval, *HTN* hypertension, *PHT* prehypertension, *TC*, total cholesterol, *TG* triglycerides

^a^Reference group

### Awareness, treatment and control of hypertension by sex

As shown in Table 3, among the 1180 hypertensive individuals, 430 were aware of their condition. The awareness of HTN was 36.5% and there was significant difference between men and women (*p* <0.001). The treatment of HTN was 19.4%, only 229 individuals with HTN reported that they were taking antihypertensive drugs to treat HTN, of which 73 (31.9%) had their BP under control, resulting in a rate of HTN control at 6.2%.

**Table 3:** Awareness, treatment and control of HTN by sex

Hypertensive cases	Total (*n* = 1180)	Women (*n* = 203)	Men (*n* = 977)	*p*-value
Awareness	430 (36.5)	50 (24.6)	380 (38.9)	< 0.001
Treatment	229 (19.4)	35 (17.2)	194 (19.9)	0.391
Control	73 (6.2)	16 (7.9)	57 (5.8)	0.271
Controlled among treated subjects	73/229 (31.9)	16/35(45.7)	57/194(29.4)	0.056

Data are presented as number (%)

*Abbreviations: HTN* hypertension

## Discussion

Our findings showed that the adjusted prevalence of HTN was 26.7%, which is comparable with some studies among Chinese adults in other regions [2, 20], ranging from 24.6 to 41.0%. In a nationally representative sample of 15 019 working-age Chinese adults, the prevalence of HTN was 23.5% [21], which is lower than the corresponding figure in this study. Similarly, the present observation was higher than the HTN prevalence in Ireland (12.8%) [11] and Hungary (22.6%) [10], while lower than that in America (29.5%) [5]. Equally important was the finding of 41.5% of subjects identified as PHT, and the adjusted prevalence of PHT was 41.3%, which is considerably higher than prior estimates for adults in China (21.9%) [4] and America (23.1%) [5]. Growing evidence suggests that individuals with PHT would have a significant higher proportion of developing to HTN and risk of CVD within a few years [10], compared to those with NTN. Considering the average age of this working population, 39.8 years, is younger than the general population, and they spend one-third of their daily activity at work. Hence, effective measures targeting at employees with PHT are urgently needed to be developed at the workplaces.

HTN and PHT, which affected more than two-thirds (69.9%) of the working population at high altitude in China, represents a major public health concern. The possible reasons for this high prevalence of HTN and PHT among young employees may be due to the high prevalence of overweight/obesity (39.1%) and smoking (43.5%), as well as the high proportion of family history of HTN (37.2%). Meanwhile, chronic exposure to high-altitude hypoxia environment may also lead to elevated BP [9].

In accordance with previous reports [10, 22], men had a higher overall prevalence rate of HTN and PHT than women, which resulted from the higher prevalence of metabolic risk factors for HTN and PHT [2, 22] (i.e., overweight or obesity, high TC, high TG, and diabetes) found in men. Our data showed that the prevalence of HTN increased with age in both men and women whereas the prevalence of PHT decreased in men older than 30 years. Moreover, there was an increase in the prevalence of PHT for women as age increased, with a peak in the prevalence of PHT for women between the ages of 50 to 59 years old, similar results are reported in adults from the United States [23]. It seems that the changing levels of hormones with age between sexes can interpret this difference [22].

In our survey, subjects with PHT had intermediate levels of BMI, TC, TG, rates of overweight or obesity, smoking, and drinking and these values increased significantly in parallel to BP. In the multivariable analysis, sex and age were significantly associated with HTN and PHT, which were confirmed in some studies [2, 22]. Overweight and obesity are important modifiable risk factors of HTN and PHT [2, 4, 24], as demonstrated in our study. When obesity coexists with elevated BP, it may further increase the development of CVD [24], thus, weight control should be prioritized for employees with high BP. Some studies have indicated that high TC and high TG are causes of HTN or PHT [2, 4, 22], and it was also noted that rising levels of TC and TG were related with HTN and PHT in our results. However, these two factors were not confirmed after adjusting for confounders. Our study showed that current smokers were at lower risk of PHT, an observation which was also described in other studies [25]. The reason for this association is still unclear and we will continue to study it. In earlier studies, alcohol use has been found to be a significant risk factor of both HTN and PHT [22, 24]. We observed that frequent drinking were predictors of HTN but not for PHT, this dichotomy was probably related with the different definitions for drinking.

An earlier study conducted in six provinces of China indicated that the prevalence of HTN varied widely among Chinese ethnic groups, and Han Chinese had higher prevalence of HTN [6]. Consistent with this trend, we found that Han Chinese had a higher risk of HTN compared with non-Han. The difference in demographic factors, lifestyle and population genetics structure of the studied population may underlie this variability [26]. Our study confirmed the findings from previous study in northeast China that high levels of education is associated with a lower risk of PHT [25]. Individuals with higher degree were supposed to have adequate HTN knowledge [27], thus they were better informed about BP management and developed healthy lifestyles subsequently. In addition, studies showed that employees belonging to higher job position had a higher risk of developing HTN and PHT [20, 25], similar conclusion was found in our study. The possible explanations are following: 1) Supervisors may suffer from higher psychosocial work stress than general staffs, and high work stress has been shown to be associated with increased BP [28]. 2) Compared with the general staffs, the supervisors usually spent more time sitting at desk and working on computers, and a decrease in physical activity may also cause the high BP.

We also analyze the relationship between family history of HTN and elevated BP. After adjusting the potential confounders, our results indicated that normotensive individuals with a family history of HTN tend to have greater risk of HTN and PHT than those without. Therefore, primary prevention strategies should be targeted at subjects with a family history. We observed that the levels of FBG and SUA, and proportion rates of diabetes and hyperuricemia increased with the elevated BP. Diabetes and hyperuricemia were independent predictors for HTN after adjusting sex, age and other risk factors, which correspond with prior studies [20, 24, 29]. This finding suggests the importance of early detection of FBG and SUA among those with elevated BP.

The rates of awareness, treatment and control of HTN in this study are lower than that previously reported in western China (36.5% vs 43.6%,19.4% vs 35.8%, 6.2% vs 9.3%) [21] and in other developed countries [30], although higher than that in the China Kadoorie Biobank Study [31]. Divergent results may partly be explained by characteristics of research including differences in region, age, occupation, etc. For instance, 87.8% of subjects in our survey were under 50 years old, working and living at less developed western China, the young population may never have measured their BP before or been told the dangers of rising BP. It is noteworthy that among those who underwent antihypertensive treatment, only about one-third achieved BP control, which was probably influenced by the medicine adherence of the hypertensive patients [15]. Moreover, the choice of antihypertensive drugs at high altitude regions can also have an impact on BP control [32].

It should be noted that this study has several limitations. First, the data came from a cross-sectional survey in a homogenous working group at high altitude, which would not be representative of the entire working population. Thus the generalizability of current reported information to other regions of China could be limited. Second, the design of cross-sectional study leaves open for interpretation of casual relationships between the development of HTN, PHT and their risk factors, which implies that our results must be interpreted with some caution. Third, the special local diet of Qinghai-Tibet plateau, including butter tea, beef and mutton, all of which containing high salts and cholesterols and may contribute to the elevation of BP [33], however, diet and salt intake was not included due to unavailable evidence-based data in our survey. Fourth, work stress might raise BP [28], while it was not investigated in this study. Finally, our results are lacking information on individual’s working altitude, as well as the changes of physiologic parameters at different working altitudes, e.g., oxygen saturation, resting heart rate, and indices of red blood cell. Those parameters are well-known to be influenced by chronic exposure to high altitude as body’s compensatory responses to hypoxia environment [34], which could be associated with BP levels. Future study is needed to examine the physiologic parameters and identify the underlying mechanisms regarding the impact of altitude on BP.

## Conclusions

This study suggests a high prevalence of HTN and PHT in a working population aged 20-59 years at high altitude in China, which may be predictive of high incidence of future CVD events. Men, higher age and job position, overweight or obesity, and family history of HTN are predictors of both HTN and PHT. Despite the high prevalence of HTN, the rates of awareness, treatment and control were unacceptable low. To tackle the challenge, workplace-based BP screening and intervention programs that aim to modify risk factors such as high BMI, tobacco use, alcohol consumption and inappropriate use of antihypertensive medicine should be conducted.

## Additional file

Additional file 1: Table S1. The prevalence rates of CVD risk factors by sex. The data provided represent the statistical analysis of the prevalence rates of CVD risk factors i.e. high BMI, smoking, drinking, high TC and TG, family history of hypertension, diabetes and hyperuricemia, for both men and women. (DOCX 13 kb)

## Abbreviations

BMI: Body mass index
BP: Blood pressure
CVD: Cardiovascular disease
DBP: Diastolic blood pressure
HTN: Hypertension
PHT: Prehypertension
SBP: Systolic blood pressure
