# Supplementary material for: Prevalence and risk factors associated with hypertension and prehypertension in a working population at high altitude in China: a cross-sectional study
Source: Environ Health Prev Med. 2017 Apr 4;22:19. doi: 10.1186/s12199-017-0634-7 (PMC5664790; doi:10.1186/s12199-017-0634-7)
Supplement: Additional file 1: Table S1. — The prevalence rates of CVD risk factors by sex. The data provided represent the statistical analysis of the prevalence rates of CVD risk factors i.e. high BMI, smoking, drinking, high TC and TG, family history of hypertension, diabetes and hyperuricemia, for both men and women. (DOCX 13 kb) [file 12199_2017_634_MOESM1_ESM.docx]

**Additional file 1: Table S1** The prevalence rates of CVD risk factors by sex

| **CVD risk factors** | **Total (n=4198)** | **Women (n=1172)** | **Men (n=3026)** | ***p*-Value** |
| --- | --- | --- | --- | --- |
| Overweight or obesity | 1643 (39.1) | 259 (22.1) | 1384 (45.7) | <0.001 |
| High TC | 120 (2.9) | 20 (1.7) | 100 (3.3) | 0.005 |
| High TG | 784 (18.7) | 104 (8.9) | 680 (22.5) | <0.001 |
| Current smoking | 1828 (43.5) | 37 (3.2) | 1791 (59.2) | <0.001 |
| Frequent drinking | 428 (10.2) | 10 (0.9) | 418 (13.8) | <0.001 |
| Family history of HTN | 1494 (35.6) | 493 (42.1) | 1001 (33.1) | <0.001 |
| Diabetes | 228 (5.4) | 24 (2.1) | 204 (6.7) | <0.001 |
| Hyperuricemia | 1094 (26.1) | 199 (17.0) | 895 (29.6) | <0.001 |

Abbreviations: TC, total cholesterol; TG, triglycerides; HTN, hypertension. Data are presented as number (%).
